# Critical Role of Hepatic Cyp450s in the Testis-Specific Toxicity of (5R)-5-Hydroxytriptolide in C57BL/6 Mice

**DOI:** 10.3389/fphar.2017.00832

**Published:** 2017-11-21

**Authors:** Cunzhi Yu, Yu Li, Mingxia Liu, Man Gao, Chenggang Li, Hong Yan, Chunzhu Li, Lihan Sun, Liying Mo, Chunyong Wu, Xinming Qi, Jin Ren

**Affiliations:** ^1^Center for Drug Safety Evaluation and Research, State Key Laboratory of Drug Research, Shanghai Institute of Materia Medica, Chinese Academy of Sciences, Shanghai, China; ^2^University of Chinese Academy of Sciences, Beijing, China; ^3^Department of Pharmaceutical Analysis, China Pharmaceutical University, Nanjing, China

**Keywords:** (5R)-5-hydroxytriptolide, functional knockout, cytochrome P450, testes, γ-H2AX, RNA polymerase II, androgen receptor

## Abstract

Low solubility, tissue accumulation, and toxicity are chief obstacles to developing triptolide derivatives, so a better understanding of the pharmacokinetics and toxicity of triptolide derivatives will help with these limitations. To address this, we studied pharmacokinetics and toxicity of (5R)-5-hydroxytriptolide (LLDT-8), a novel triptolide derivative immunosuppressant in a conditional knockout (KO) mouse model with liver-specific deletion of CYP450 reductase. Compared to wild type (WT) mice, after LLDT-8 treatment, KO mice suffered severe testicular toxicity (decreased testicular weight, spermatocytes apoptosis) unlike WT mice. Moreover, KO mice had greater LLDT-8 exposure as confirmed with elevated AUC and Cmax, increased drug half-life, and greater tissue distribution. γ-H2AX, a marker of meiosis process, its localization and protein level in testis showed a distinct meiosis block induced by LLDT-8. RNA polymerase II (Pol II), an essential factor for RNA storage and synapsis in spermatogenesis, decreased in testes of KO mice after LLDT-8 treatment. Germ-cell line based assays confirmed that LLDT-8 selectively inhibited Pol II in spermatocyte-like cells. Importantly, the analysis of androgen receptor (AR) related genes showed that LLDT-8 did not change AR-related signaling in testes. Thus, hepatic CYP450s were responsible for *in vivo* metabolism and clearance of LLDT-8 and aggravated testicular injury may be due to increased LLDT-8 exposure in testis and subsequent Pol II reduction.

## Introduction

Triptolide is a structurally unique diterpenoid from *Tripterygium wilfordii* Hook F, and has excellent efficiency against cancers, polycystic kidney disease and rheumatic disease (Leuenroth and Crews, 2008; Zheng et al., 2008; Leuenroth et al., 2010; Mujumdar et al., 2010; Pan, 2010; Liu, 2011; Liu et al., 2011, 2013a,b; Manzo et al., 2012; Kim et al., 2014; Lu et al., 2014; Sangwan et al., 2015; Fan et al., 2016). By inhibiting XPB via covalent binding, a DNA helicase and a component of the TFIIH transcription complex, triptolide induced transcription repression and Pol II degradation in cancer cells (Titov et al., 2011; Chen et al., 2015). Other mechanisms such as Hsp70 inhibition, JNK and NF-kB signal pathways may also play a role (Villicana et al., 2013; Sangwan et al., 2015; Zhang et al., 2016). Triptolide inhibits dCTP pyrophosphatase activity, converting CTP to dCMP with a non-covalent interaction, and attenuates cystic kidney disease (Corson et al., 2011).

Lack of aqueous solubility, general toxicity, and tissue accumulation limited the clinical use of triptolide (Ye et al., 2010; Sun et al., 2013; Zhuang et al., 2013; Kong et al., 2015; Ma et al., 2015; Patil et al., 2015; Qi et al., 2015; Wang et al., 2016a,b, 2017; Ruan et al., 2017). Overexposure and subsequent toxicity of triptolide lead to the clinical trial termination of F6008, a prodrug of triptolide (Fidler et al., 2003; Kitzen et al., 2009). While the incomplete cleavage, loss of drug bioactivity and interpatient variability stopped the clinical trials of other prodrugs of triptolide, PG490-88, and omtriptolide (Kitzen et al., 2009). Therefore, clarifying the connection between toxicity and pharmacokinetics of triptolide and its derivatives may help us overcome some limitations of triptolide and allow the clinical use of triptolide and its derivatives (Zhou et al., 2012).

(5R)-5-hydroxytriptolide (LLDT-8) is a novel triptolide derivative with potent immunosuppressive, anti-inflammatory, and anticancer activity (Zhou et al., 2005; Wang et al., 2012; Zeng et al., 2016; Su et al., 2017), and it is now in phase II clinical trials in China for the treatment of rheumatoid arthritis (Qi et al., 2016). LLDT-8 reduces the production of Th1 type cytokines (IFN-γ, IL-2) and inflammatory cytokines (TNF-α, IL-16), and inhibits NF-kB activation triggered by lipopolysaccharide (Zhou et al., 2006a,b). LLDT-8 also has potent anticancer activity via transcription inhibition (Wang et al., 2012).

Compared to triptolide (Xue et al., 2011), LLDT-8 has a better safety profile and does not induce abnormalities in the epididymis, liver, kidney, spleen, or circulation (Qi et al., 2016). The testicular injury is the main adverse effect of LLDT-8 in rodents, and recently we reported that spermatocytes are the primary target for LLDT-8 in the testes. Dephosphorylating TGF-β activated kinase (Tak1) Ser412 contributes to this selectivity (Qi et al., 2016). However, the interaction between toxicity and pharmacokinetics of LLDT-8 remained unknown.

The functional redundancy of CYP450 makes it difficult to determine the isoforms involved in drug metabolism and toxicity. Wu et al. developed a liver-specific knockout mouse model of cytochrome P450 reductase (Cpr), the sole electron donor of CYP450s, to overcome these limitations and reduced almost 95% hepatic CYP450 activity (Wu et al., 2003). Cpr knockout inhibited the hepatic P450-dependent metabolism of monocrotaline, aristolochic acid, and triptolide (Xiao et al., 2008; Xue et al., 2011; Yao et al., 2014).

Here, this study compared the toxicity and tissue distribution of LLDT-8 between Cpr knockout and wildtype mice. Inactivation of hepatic cyp450s increased the exposure of LLDT-8 and blocked meiosis in the testes by selectively downregulating γ-H2AX and RNA polymerase II in spermatocytes.

## Materials and methods

### Chemicals

(5R)-5-hydroxytriptolide (LLDT-8, 99.9%) was from by Professor Yuanchao Li (Shanghai Institute of Materia Medica, Shanghai, China). All other chemicals were commercially available and purchased as reagent grade from Sigma-Aldrich (St. Louis, USA). The following antibodies were used for western blotting: γ-H2AX (Cat. No: 2577, Lot. No: 11, CST, USA), RNA polymerase II (Cat. No: 05-623, Lot. No: 2397109, Millipore, USA), β-actin (Cat. No: sc-47778, Lot. No: 2533, Santa Cruz, USA). γ-H2AX (Cat. No: ab26350, Lot. No: GR305763-3, Abcam, USA) was used for immunofluorescence assay.

### Animal treatments

Animal use protocols were approved by the Institutional Animal Care and Use Committee of the Shanghai Institute of Materia Medica (Shanghai, China), IACUC No. 2016-10-RJ-136. The Cpr knockout (KO) mice were a gift from Professor Xinxin Ding (Wadsworth Center, Albany, NY, USA). Procedures for animal breeding and genotyping were reported previously (Wu et al., 2005). Eight to twelve weeks old male KO mice and their WT littermates on a mixed C57BL/6 and 129/Sv genetic background were used in this study. All animals were provided with a certified standard diet and tap water *ad libitum* during experiments. All animals were maintained under controlled temperature with a 12 h:12 h light/dark cycle. All efforts were made to minimize animal discomfort and illness, and mice were anesthetized with pentobarbital sodium (150 mg/kg, i.p.) before sample collection. No animals died during the experiment.

### Toxicological study

The LD_50_ of LLDT-8 was 9.3 mg/kg (p.o.) in mice (Zhou et al., 2005), and we used a more clinically relevant dose in our animal experiments. For toxicity experiments, mice (*n* = 3/group) were treated with LLDT-8 (0.5 or 1.0 mg/kg) consecutively administered by gavage for 15 days. Controls received saline. Mice were killed on the 15th-day post-administration and blood, liver, kidney, spleen, testes, and epididymis were collected. About 500 μL blood samples were collected from the heart. The main lobe of the liver, kidney, spleen, and epididymis was fixed in 10% neutral buffered formalin for histological examination, and the left testicle was fixed in modified Davidson's buffer (30% of a 37–40% solution of formaldehyde, 15% ethanol, 5% glacial acetic acid, and 50% distilled H_2_O) for 16 h followed by 10% neutral buffered formalin for 24 h (Latendresse et al., 2002). Tissue sections were stained with hematoxylin and eosin (H&E). Remaining tissues were stored at −80°C for RNA and protein extraction.

Sera were assayed for Urea, creatine (CRE), alanine aminotransferase (ALT), and aspartate transaminase (AST) using an automatic HITACHI Clinical Analyzer Model 7080 (Hitachi High-Technologies Corporation, Tokyo, Japan). The intercoefficients of variability in this assay were1.1% (ALT), 0.9% (AST), 1.2% (Urea) and 1.4% (CRE). The intracoefficients of variability in this assay were 2.0% (ALT), 0.9% (AST), 1.2% (Urea) and 5.1% (CRE).

### Toxicokinetics of LLDT-8 in mice

For toxicokinetic experiments, mice (*n* = 5–6/group for each time point) were treated with a single dose of LLDT-8 at (0.5 or 1.0 mg/kg) by oral gavage, creating 4 treatment groups: 0.5 mg/kg LLDT-8-treated WT and KO mice, and 1.0 mg/kg LLDT-8-treated WT and KO mice. After LLDT-8 treatment, 20 μL blood samples were collected at 5, 10, 20, 30, 45, 60, 90, 120, and 180 min from tail vein. Plasma was separated by centrifugation at 3,000 rpm for 5 min and kept at −80°C until analysis. Tissues including the liver, kidney, testes, and epididymis were also collected from individual mice at 30 and 180 min after dosing and were weighed and homogenized in saline (1.0 g wet weight/mL) on ice. LLDT-8 was then extracted from the plasma or the tissue homogenates with an equal volume of ethyl acetate and dried under nitrogen. The residues were reconstituted in 50 μL of mobile phase for analysis.

The quantification of LLDT-8 in samples was performed on Shimadzu 20A HPLC system (Shimadzu, Kyoto, Japan) equipped with an autosampler coupled to Shimadzu 8030 triple quadrupole mass spectrometer (Shimadzu, Kyoto, Japan). Separations were conducted under isocratic conditions. The mobile phase consisting of acetonitrile and water with 0.1% formic acid (50:50, v/v) was set at a flow rate of 0.2 mL/min. An electrospray interface in negative ionization mode was used. ESI source parameters were as followed: high purity drying-gas (N2) flow rate 8 L/min, temperature 400°C, nebulizer pressure 25 psi. Multiple reaction monitoring (MRM) was used to quantify LLDT-8 (m/z 421.20 [M+COO]- → 45.10, fragmentor 110 eV, collision energy −20 eV). Analytical data were processed using the labsolution software package (Shimadzu, Kyoto, Japan) consisting of qualitative and quantitative software.

Standard curves for LLDT-8 were prepared by spiking known amounts of the LLDT-8 standard into plasma or tissue homogenate samples prepared from untreated mice. LLDT-8 concentrations in biological samples were determined by comparisons with standard curves. Pharmacokinetics were calculated using Kinetica software (version 4.4.1; Thermo Fisher Scientific Inc, Woburn, MA).

### Metabolic profile of LLDT-8

Metabolic profiles of LLDT-8 in liver microsomes from WT and KO mice were compared. In brief, 1 mM NADPH (Cat. No: 10107824001, Lot. No: 20595625, Sigma, USA), 5 mM MgCl_2_, 10 μL mouse liver microsomes (20 mg/mL, microsomes were prepared from the liver of mouse pretreated with 80 mg/kg dexamethasone for 3 days), 4 μL LLDT-8 (12.5–50 μM) in a 100 mM phosphate buffer (pH 7.4). There was a 3 min preincubation step at 37°C before initiating the reaction by adding the NADPH into the microsomal suspension. The reaction was stopped with ice-cold acetonitrile after 60 min incubation. Identification of LLDT-8 metabolites was performed on an Agilent 1200 HPLC system (Agilent technologies Inc, Palo Alto, CA) equipped with a CTC PAL autosampler coupled to an API4000 QTRAP LC-MS/MS system (Thermo Fisher Scientific, USA). Separations were conducted under gradient conditions. The mobile phase consisting of 0.1% formic acid in acetonitrile and 0.1% formic acid in water (50:50, v/v) was set at a flow rate of 0.6 mL/min. An electrospray interface in negative ionization mode was used. ESI source parameters were as followed: high purity drying-gas (N_2_) flow rate 8 L/min, temperature 550°C, and nebulizer pressure 25 psi. Single ion monitoring with EMS/EPI scan mode was used to quantify LLDT-8 (positive: m/z 377.1, collision energy 60 eV; Negative: m/z 375.1, collision energy 60 eV; negative: m/z 375.1), and mono-hydroxylated LLDT-8 was selected with EPI scan mode (CE: −45 v, CES: −15).

### Chemical inhibition study

The chemical inhibition assay was performed as previously reported (Li et al., 2008). A 400 μl typical incubation mixture contained 0.2 mg rat liver microsomes (20 mg/ml, Cat. No: LM-DS-02M, Lot. No: BDVH, Research Institute for liver Diseases, Shanghai, China), 2 μM LLDT-8, 5 mM MgCl_2_, 1 mM NADPH (Cat. No: 10107824001, Lot. No: 20595625, Sigma, USA) and selective inhibitors of each CYP isoforms in a 100 mM phosphate buffer (pH 7.4). The inhibitors used were as follows: quercetin (2 μM) for CYP1A and CYP2C8, 8-methoxypsoralen (2.5 μM) for CYP2A6, sulfaphenazole (10 μM) for CYP2C9, omeprazole (20 μM) for CYP2C19, quinidine (10 μM) for CYP2D6, clomethiazole (50 μM) for CYP2E1, ketoconazole (1 μM) for CYP3A4 and aminobenzotriazole (50 μM, a broad CYP inhibitor) (Tsyrlov et al., 1994; Li et al., 2008). After incubation for 0, 0.5, and 1 h, 100 μl supernatant was collected and quenched by the addition of 100 μl of ice-cold acetonitrile. The mixtures were then centrifuged for 10 min at 20,000 × g. An aliquot of the supernatant was analyzed by LC-MS/MS to monitor the residual LLDT-8 without extraction.

### Cell culture

Mouse GC-1spg (spermatogonia-like, ATCC number: CRL-2053), TM4 (Sertoli cell-like, ATCC number: CRL-1715), and GC-2spd (spermatocyte-like, ATCC number: CRL-2196) were purchased from ATCC (Manassas, VA). Cells were grown in a 5% CO_2_ atmosphere at 37°C. TM4 was cultured in DMEM supplemented with 5% horse serum, 2.5% fetal bovine serum and 1x antibiotic-antimyotic (Cat. NO. 15240062, Life technologies). GC-1spg and GC-2spd were cultured in DMEM supplemented with 10% fetal bovine serum (Cat. No. F2442, Sigma, USA) and 1x antibiotic-antimyotic (Cat.NO. 15240062, Life technologies).

### Quantitative real-time PCR (qPCR)

Total mouse testes RNA was isolated using a UNIQ-10 total RNA isolation kit (Sangon Biotech, Shanghai, China). One micro gram RNA per sample was reverse-transcribed into cDNA using a PrimeScript RT reagent kit (Cat. No. RR036A, Takara, Dalian, China). The purity and quality of RNA and cDNA were checked by A260/A280 ratio and agarose gel. The qPCR was executed in 20 μL volume containing 10 μL SYBR Premix Ex Taq (Cat. No. RR820A, Takara, Dalian, China), 8 μL of cDNA (80 ng cDNA), 1 μL forward primer (500 nM) and 1 μL reverse primer (500 nM). The sequences of primers for Androgen-binding protein (ABP) and cystatin 12 (Cst12) were referenced from PrimerBank. The sequences for other genes were described elsewhere [Deleted in azoospermia-like (Dazl), Heat shock protein a2 (Hspa2), Phosphoglycerate kinase-2 (Pgk2), Protamine 1 (Prm1), GATA binding protein 1 (Gata1) and Zbtb16 zinc finger and BTB domain containing 16 (Plzf) (Qi et al., 2016); AR (Ma et al., 2013); Reproductive homeobox 5 (Rhox5) (Kurek et al., 2015); Fatty acid binding protein (FABP) (Vanschoonbeek et al., 2008); Claudin 11 (Cldn11) (Sumigray et al., 2014); β-actin (Wan et al., 2017)]. All the sequences of primers were listed in Table 1. The qPCR process was performed using a Rotor Gene Q PCR system (QIAGEN, Shanghai, China). The qPCR amplification program consisted of polymerase activation at 98°C for the 30 s and 40 cycles of denaturation at 95°C for 5 s, annealing, and extension at 60°C for 40 s. The melting curve analysis was carried out for each reaction from 50 to 99°C. The CT values for the samples were normalized to the corresponding β-actin CT values.

**Table 1:** Primers sequences for qPCR.

**Gene**	**Accession number**	**Primer sequences (5′-3′)**
Dazl	NM_010021	Forward:CCTCCAACCATGATGAATCC
		Reverse: TCTGTATGCTTCGGTCCACA
Hspa2	NM_008301	Forward: CATCATCAATGAGCCCACAG
		Reverse:TCTTGTGTTTGCGCTTGAAC
Pgk2	NM_031190	Forward: CTGTTGCTGATGAGCTCAAG
		Reverse: ACTCCGACCATAGAACTGTG
Prm1	NM_013637	Forward: ATGCTGCCGCAGCAAAAGCA
		Reverse: CACCTTATGGTGTATGAGCG
Gata1	NM_008089	Forward: CAGGTTTCTTTTCCTCTGGG
		Reverse: AAAGGACTGGGAAAGTCAGC
Plzf	NM_001033324	Forward: TGAGATCCTCTTCCACCGAA
		Reverse: GTAGGACTCATGGCTGAGAGA
AR	NM_013476	Forward: CTGGGAAGGGTCTACCCAC
		Reverse: GGTGCTATGTTAGCGGCCTC
Rhox5	NM_008818	Forward: ACTCGGAAGAACAGCATGATG
		Reverse: CCCTGGTGCCACTATCCTT
ABP	NM_011367	Forward: TCTGCTGTTGCTACTACTGATGC
		Reverse: GGGCCATTGCTGAGGTACTTA
FABP	NM_024406	Forward: AAGGTGAAGAGCATCATAACCCT
		Reverse: TCACGCCTTTCATAACACATTCC
Cst12	NM_027054	Forward: CGTGTTCCACTTCAACGAAAAC
		Reverse: GCCCATCTCCAGGTCTACTAAAT
Cldn11	NM_008770	Forward: ATGGTAGCCACTTGCCTTCAG
		Reverse: AGTTCGTCCATTTTTCGGCAG
β-actin	NM_007393	Forward: GCATTGCTGACAGGATGCAG
		Reverse: GAGCCACCGATCCACACAGA

### Western blot analysis

Cells were washed twice with ice-cold PBS (137 mM NaCl, 2.7 mM KCl, 10 mM Na_2_HPO4, and 1.8 mM KH_2_PO4, pH 7.4) and lysed in SDS sample buffer. Cell lysates, containing 20 μg of protein, were separated with SDS-PAGE (8%) and transferred to polyvinylidene difluoride membranes. After blocking in 5% non-fat milk in tris-buffered saline containing 0.1% Tween-20 (pH = 7.6), membranes were incubated with the appropriate primary antibodies at 4°C overnight. The primary antibodies γ-H2AX (Cat. No: 2577, Lot. No: 11, CST, USA) and Pol II (Cat. No: 05-623, Lot. No: 2397109, Millipore, USA) diluted 1:1000 in 5% bovine serum albumin (BSA, Cat. No. B2064-50G, Sigma, USA). Then the membranes were exposed to secondary antibodies (1:10,000, Cat. No.111-035-003, 115-035-003, Jackson ImmunoResearch Laboratories, USA) in 5% non-fat milk for 1 h at room temperature. Immunoreactive proteins were visualized using an enhanced chemiluminescent system (Cat. No. WBKLS0500, Millipore, Shanghai, China).

### TUNEL

TUNEL assays were performed according to the manufacturer's protocol (*In Situ* Cell Death Kit, Roche Diagnostics, Indianapolis, IN). In brief, the paraffin-embedded testis tissue samples were deparaffinized three times in exchanges wash of xylene. The testis sections were then gradually rehydrated using decreasing ethanol concentrations (100, 95, 90, 80, and 70%) followed by PBS (pH = 7.4). After digestion with proteinase K (15 μg/mL, Cat. No. 539480, Lot. No. D00148754, Millipore, USA), paraffin-embedded tissue sections were labeled with a TUNEL reaction mixture, which contained terminal deoxynucleotidyl transferase incorporated with fluorescein. And 1 μg/mL DAPI (Sigma, USA) to used to reveal nuclear DNA. After staining, images were obtained with a fluorescent microscope (NIKON TS2, Japan).

### Immunofluorescence

For immunofluorescence analysis, the paraffin-embedded testis tissue samples were deparaffinized three times in exchanges wash of xylene. The testis sections were then gradually rehydrated using decreasing ethanol concentrations followed by PBS (pH = 7.4). After antigen retrieval in boiling 0.1M citrate buffer (pH = 6.0), the tissue sections were placed in humidity chambers and incubated with 4% BSA in PBS for 2 h at room temperature. Then 1:400 1% BSA diluted γ-H2AX primary antibody (Cat. No: ab26350, Lot. No: GR305763-3, Abcam, USA) and isotope control Mouse IgG1 (Cat. No: sc-3877, Lot. No: F1316, Santa Cruz, USA) was used. After incubated with primary antibody overnight at 4°C, testis tissue samples were washed three times with PBS. Then the samples were incubated with 1:500 diluted secondary antibody (Alexa Fluor 594 (red)-conjugated Affinipure Donkey Anti-mouse IgG (Cat. No. 715-585-150, Life Technologies, USA) for 0.5 h at room temperature. And 1 μg/mL DAPI (Sigma, USA) to used to reveal nuclear DNA. After mounted in an antifade solution (Cat. No. S36963, Life Technologies, USA), tissue samples were observed using a confocal microscope (LEICA, USA).

### Statistical analysis

Statistical analysis was performed using two-tailed Student's *t*-tests or one-way ANOVA followed by LSD's *post hoc* test (*p* < 0.05 was considered statistically significant).

## Results

### Knockout of hepatic Cpr aggravated testicular injury induced by LLDT-8

**Figure 1:**
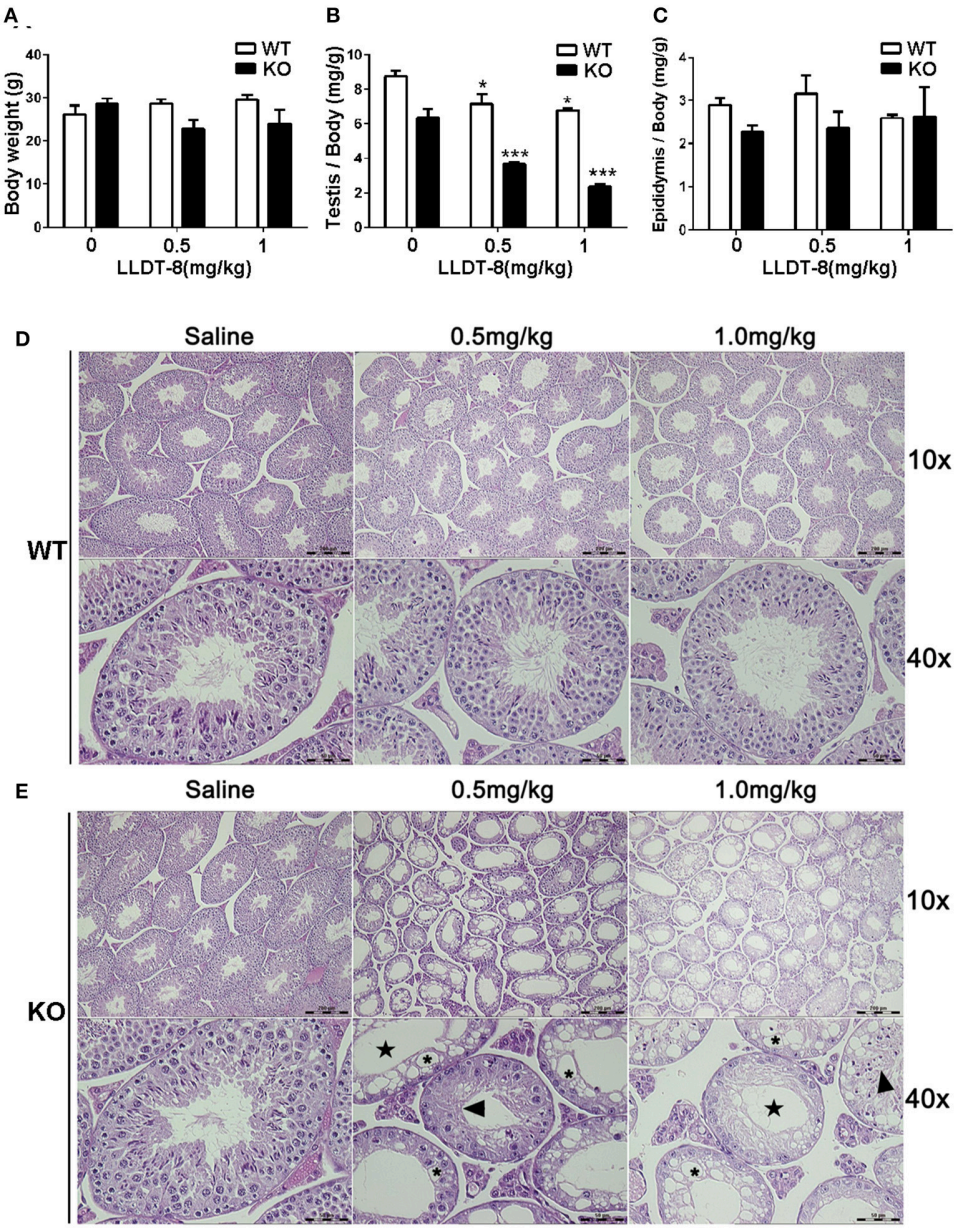
Hepatic Cpr knockout aggravated LLDT-8 induced testicular injury**. (A)** Body weights of WT and KO mice; **(B)** Testis relative weight (absolute testis weight vs. body weight); **(C)** Epididymis relative weight (absolute epididymis weight vs. body weight); H&E sections of the left testicle of WT **(D)** and KO **(E)** mouse (× 10, × 40); Star: reduction of germinal layers; Asterisk: vacuolar degeneration; Arrow: abnormally developed spermatids. Significant difference was determined by one way ANOVA, mean ±*SD, n* = 3, ^*^p <0.05, ^***^p <0.001 vs. Saline group.

**Figure 2:**
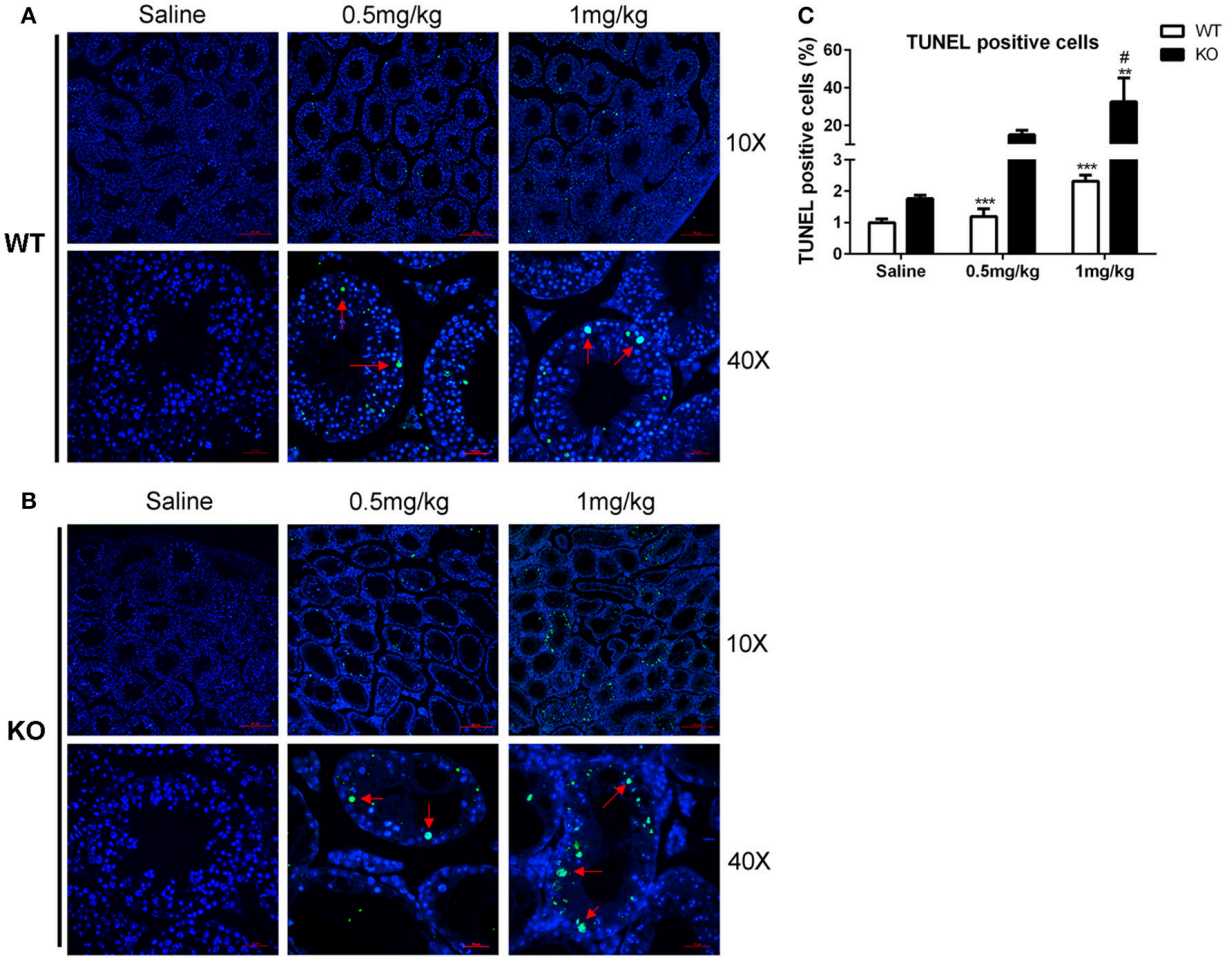
TUNEL assay of LLDT-8 induced testis injury. The paraffin-embedded testis sections of WT **(A)** and KO **(B)** mice were labeled with TUNEL reaction mixture to distinguish the TUNEL positive cell (× 10, × 40). Arrow: TUNEL positive cell. **(C)** Quantification of TUNEL positive cells. A significant difference was determined by one way ANOVA, mean ± *SD, n* = 3, ^**^p < 0.01, ^***^p < 0.001 vs. Saline group, #*p* < 0.05 vs. WT mice at the same dosage.

The dosage of LLDT-8 (0.5–1 mg/kg) in this study was clinically relevant. Chronic administration of LLDT-8 did not alter the body weight of WT and KO mice, but dose-dependently reduced the testes (Figures 1A,B). The epididymis weight was not changed by LLDT-8 treatment LLDT-8 treatment (Figure 1C). Compared to WT mice, the testes in KO mice showed reduced germinal cell layers (Figures 1D,E, Star), severe vacuolar degeneration (Figure 1E, asterisk) and absence of spermatids development (Figure 1E, arrow). TUNEL assay found the number of TUNEL positive foci in KO mice testis were much higher than that in WT mice (Figures 2A,B) after LLDT-8 weight after LLDT-8 treatment in WT mice, consistent with another experiments using C57BL/6 mice (Supplementary Figure S1A). Noticeably, compared to WT mice with saline, TUNEL-positive foci in the testes of WT mice with LLDT-8 treatment apparently increased (Figures 2A,C).

LLDT-8 also induced hematological change and enlarged spleen in KO mice (Supplementary Figure S2). LLDT-8 did not cause liver injury and kidney damage (Supplementary Figures S3, S4).

### Inactivation of hepatic CYP450 increased the exposure of LLDT-8

**Figure 3:**
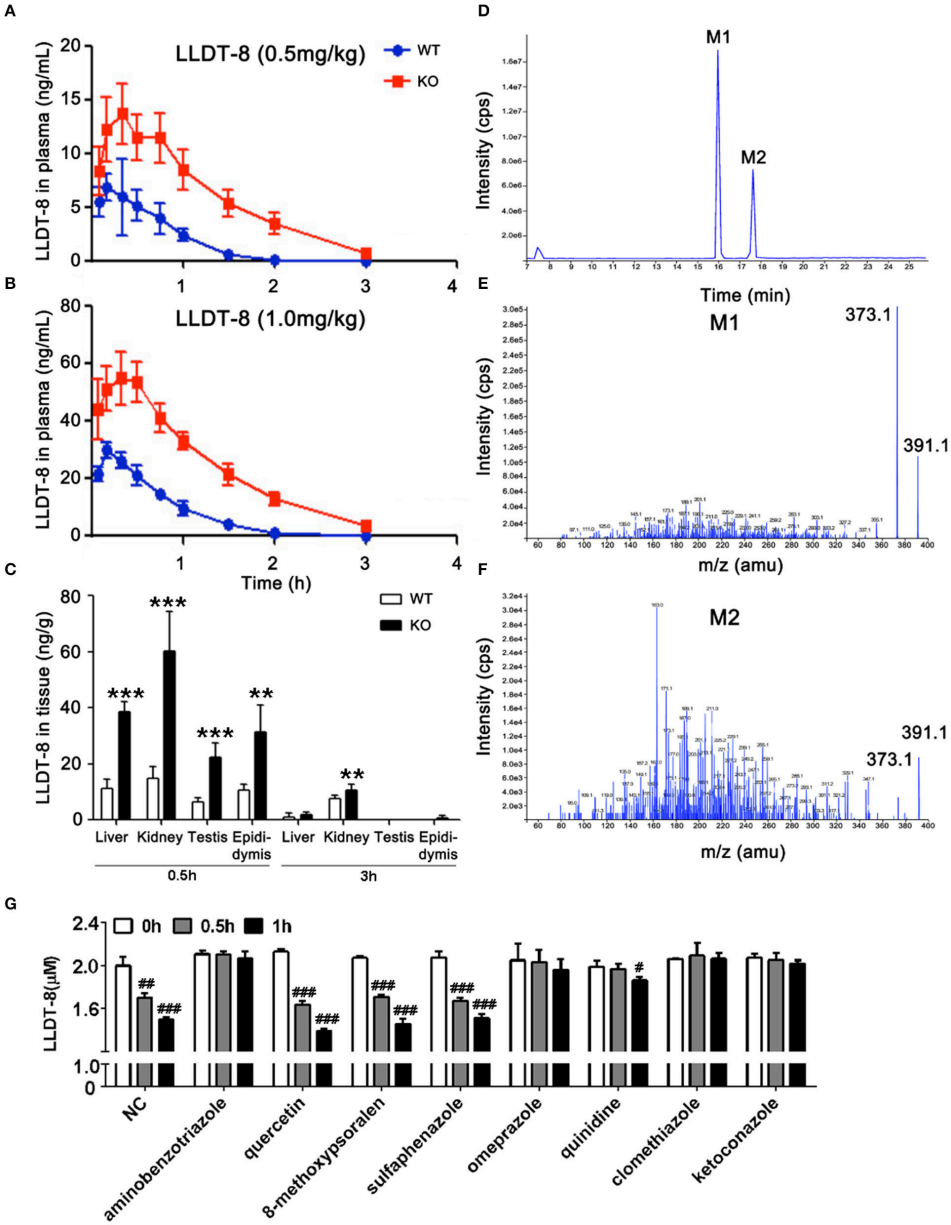
Levels of LLDT-8 in the blood, liver, kidney, testis, and epididymis of KO and WT mice following a single oral dose of LLDT-8. Levels of LLDT-8 in the plasma from 0.5 mg/kg **(A)** and 1.0 mg/kg **(B)** group, and the LLDT-8 level in the liver, kidney testis, and epididymis **(C)** were determined by LC–MS/MS. ND, not detectable; mean ± *SD, n* = 5 for each time point, ^**^p < 0.01, ^***^p < 0.001 vs. WT 1.0 mg/kg, *t*-test. **(D)** LLDT-8 metabolites (M1 and M2, m/z 391.1) were detected in the microsomal samples from WT mice. Mass spectrometry of metabolite M1 **(E)** and M2 **(F)** were characterized by fargments m/z 391.1-373.1. The retention time and peak area of each metabolite were summarized in Table 3. Inhibitory effects of CYP inhibitors on the metabolism of LLDT-8 in rat liver microsomes **(G)**. LLDT-8 (2 μM) was incubated with rat liver microsomes for 0.5 and 1 h, the residual LLDT-8 was analyzed by LC-MS/MS, mean ± *SD*, all incubations were carried out in three independent experiments in triplicate. ^#^*p* < 0.05, ^##^*p* < 0.01, ^###^*p* < 0.001 vs. 0 h.

WT and KO mice were treated with a single dose of LLDT-8 at 0.5/1.0 mg/kg by oral gavage. There is a significant increase in half-life (1.9 folds to WT mice), C_max_ (1.9 folds to WT mice) and AUC (3.1 folds to WT mice) of LLDT-8 in KO mice, accompanied by a marked decrease in the clearance, compared to those in WT mice (Figures 3A,B; Table 2). LLDT-8 levels in liver, kidney, testis, and epididymis of KO mice were about 2–3 folds higher than those of WT mice at 30 min following a dose of LLDT-8 treatment at 1.0 mg/kg (Figure 3C).

**Table 2:** Pharmacokinetic parameters for plasma LLDT-8 in WT and KO mice.

		**T1/2(h)**	**T_max_ (h)**	**C_max_(ng/ml)**	**AUC ([ng/ml] × h)**	**CL(L/kg/h)**
LLDT-8	WT	0.31 ± 0.03	0.17 ± 0.00	29.95 ± 2.81	241.48 ± 30.64	4.13 ± 0.53
	KO	0.59 ± 0.06^***^	0.33 ± 0.11^**^	57.97 ± 8.28^***^	753.52 ± 76.39^***^	1.33 ± 0.12^***^

*Plasma LLDT-8 levels after the administration of 1.0 mg/kg LLDT-8 were used to calculate pharmacokinetic parameters, including C_max_ (the maximum concentration), Tmax (time to reach C_max_), AUC (area under the curve), T1/2 (the elimination half-life) and CL (oral clearance). Data are mean ±SD, n = 5*.

** *p < 0.01*,

*** *p < 0.00.1 vs. WT group*.

Hepatic microsomes isolated from the liver of WT and KO mice were used to identify the potential metabolites of LLDT-8. Based on our previous findings on triptolide (Xue et al., 2011), we hypothesized hydroxylation is an elimination route of LLDT-8 *in vivo*. After 60 min-incubation, the recovered samples were subjected to API400 QTRAP LC-MS/MS system, single ion monitoring (SIM) with EMS/EPI scan mode was used to identify LLDT-8 (Negative: m/z 375.1), mono-hydroxylated LLDT-8 (Negative: m/z 391.1). Two mono-hydroxylated metabolites M1 and M2 (m/z = 391.1) were identified in the microsomal samples from WT mice, but not in those of KO mice (Figures 3D–F). The response of mass spectrometric response of M1 and M2 increased with the concentration of LLDT-8 (Table 3). There were no di-hydroxylated metabolites of LLDT-8 (m/z = 407.1) were identified in both WT and KO mice.

**Table 3:** Metabolite characteristics and abundances of LLDT-8 generated in liver microsomal incubations.

**Metabolite**	**m/z**	**Retention Time (min)**	**Metabolite peak ia (counts)**		
			**LLDT-8 (50 μM)**	**LLDT-8 (25 μM)**	**LLDT-8 (12.5 μM)**
M1	391.1	15.98	1.47E+08	6.63E+07	4.69E+07
M2	391.1	17.63	6.54E+07	3.61E+07	1.59E+07
LLDT-8	375.1	18.49	–	–	–

*Potential mono-hydroxylated metabolites (m/z 391.1) were identified by single-ion monitor method with API4000 QTRAP LC-MS/MS. The samples recovered from the incubation of LLDT-8 in liver microsomes from WT and KO mice were subjected to LC-MS/MS. The retention time and peak area of each metabolite were summarized here. M1 and M2 were detected at three LLDT-8 concentration levels, and their mass spectrometric response levels (Metabolite peak area) increased with increasing LLDT-8 concentration (12.5–50 μM)*.

To identify the CYP isoforms responsible for the metabolism of LLDT-8, various selective inhibitors of CYPs were employed (Figure 3G). The metabolism of LLDT-8 was inhibited by aminobenzotriazole (a broad CYP inhibitor), omeprazole (CYP2C19 inhibitor), quinidine (CYP2D6 inhibitor), clomethiazole (CYP2E1 inhibitor) and ketoconazole (CYP3A4 inhibitor) in rat liver microsomes.

### LLDT-8 decreased the expression and changed the distribution of γ-H2AX, a marker of meiosis

**Figure 4:**
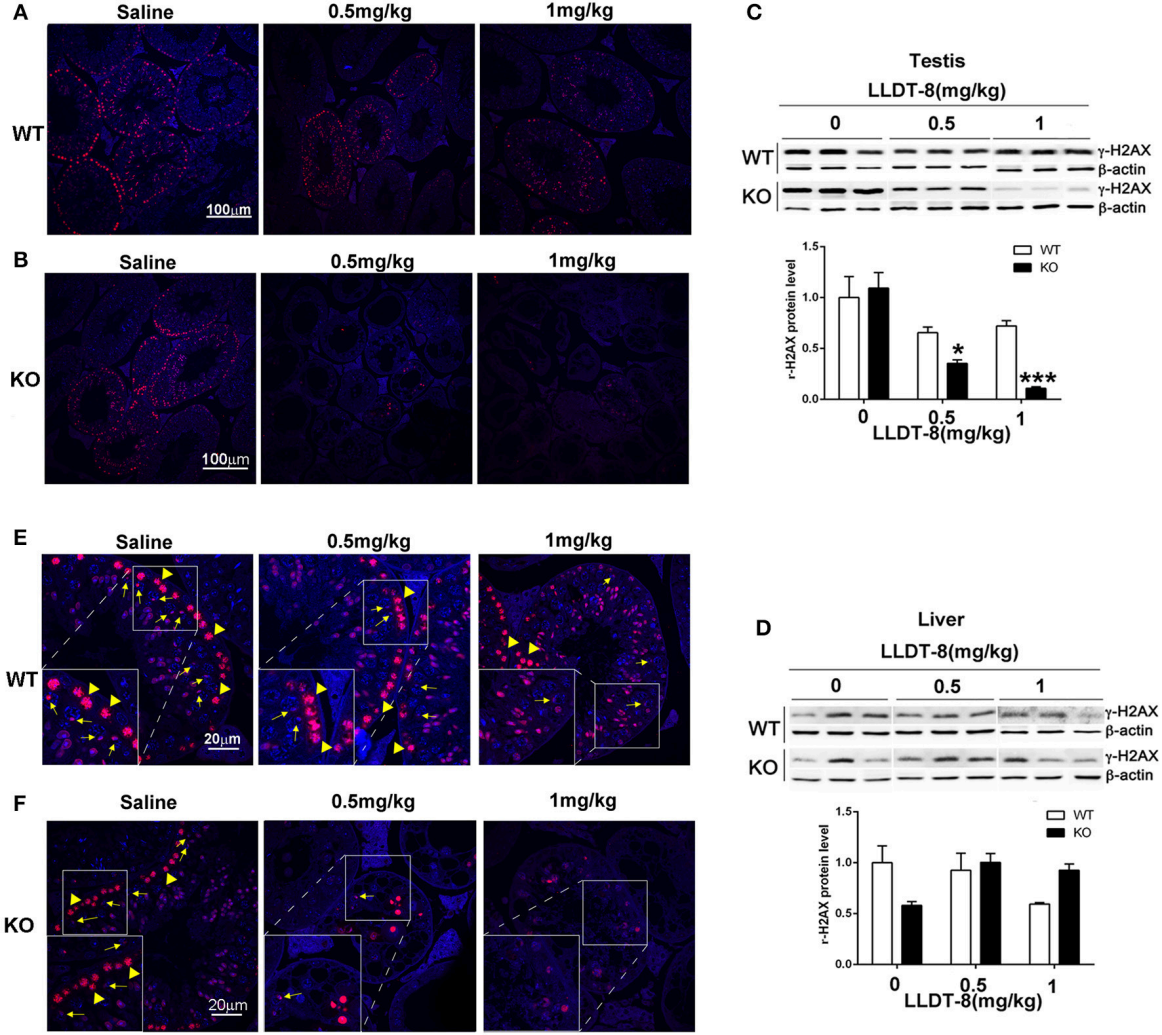
LLDT-8 dose-dependently decreased γ-H2AX in testis but not in liver. The localization of γ-H2AX in the testes of WT **(A,E)** and KO **(B,F)** mice were shown (**A,B** ×20, Scale bar: 100 μm; **E,F** ×63, Scale bar: 20 μm); The protein level of γ-H2AX in the testes **(C)**, liver **(D)** of WT and KO mice. Yellow arrow heads indicated leptotene or zygotene spermatocytes marked by the nuclei distribution of γ-H2AX, and the yellow arrows pointed the XY bodies in the process of meiosis. The protein level was quantified with ImageQuant software **(C,D)**. Beta-Actin was used as a loading control. Significant difference was determined by one way ANOVA, mean ± *SD, n* = 3, ^*^p < 0.05, ^***^p < 0.001 vs. Saline group (0 mg/kg).

To clarify the potential mechanisms of LLDT-8 induced testicular injury in KO mice, we examined the expression and distribution of γ-H2AX, a marker of meiosis and XY body formation in the testes of mice (Hamer et al., 2003; Noguchi et al., 2008; Ahmed et al., 2013). In WT and KO mice with saline, the nuclei distribution of γ-H2AX indicated the presence of leptotene or zygotene spermatocytes, characterized by the ring-like property of seminiferous tubules, which was reduced in WT mice or even disappeared in the testes of KO mice after LLDT-8 treatment (Figures 4A,B). The XY bodies indicated by γ-H2AX foci (cloud like, yellow arrow) were normal in the testes of WT and KO mice with saline, while decreased in the testes of WT mice with 1.0 mg/kg LLDT-8 and disappeared in the testes of KO mice treated with LLDT-8 (Figures 4E,F). Immunoblotting also confirmed the reduction of γ-H2AX protein level in the testes but not in the liver (Figures 4C,D). These results strongly suggested that accumulated LLDT-8 may induce a severe meiotic block in the testes of KO mice.

We also detected other germ cell markers in the testes of WT and KO mice (Supplementary Figures S5, S6) and found a marked decrease of Dazl (a marker for type B spermatogonia and primary spermatocytes), Prm1 (a marker for spermatids) and a mild decrease of Hspa2 in the testes of WT mice (Supplementary Figure S5).

### LLDT-8 selectively decreased RNA polymerase II of spermatocytes

**Figure 5:**
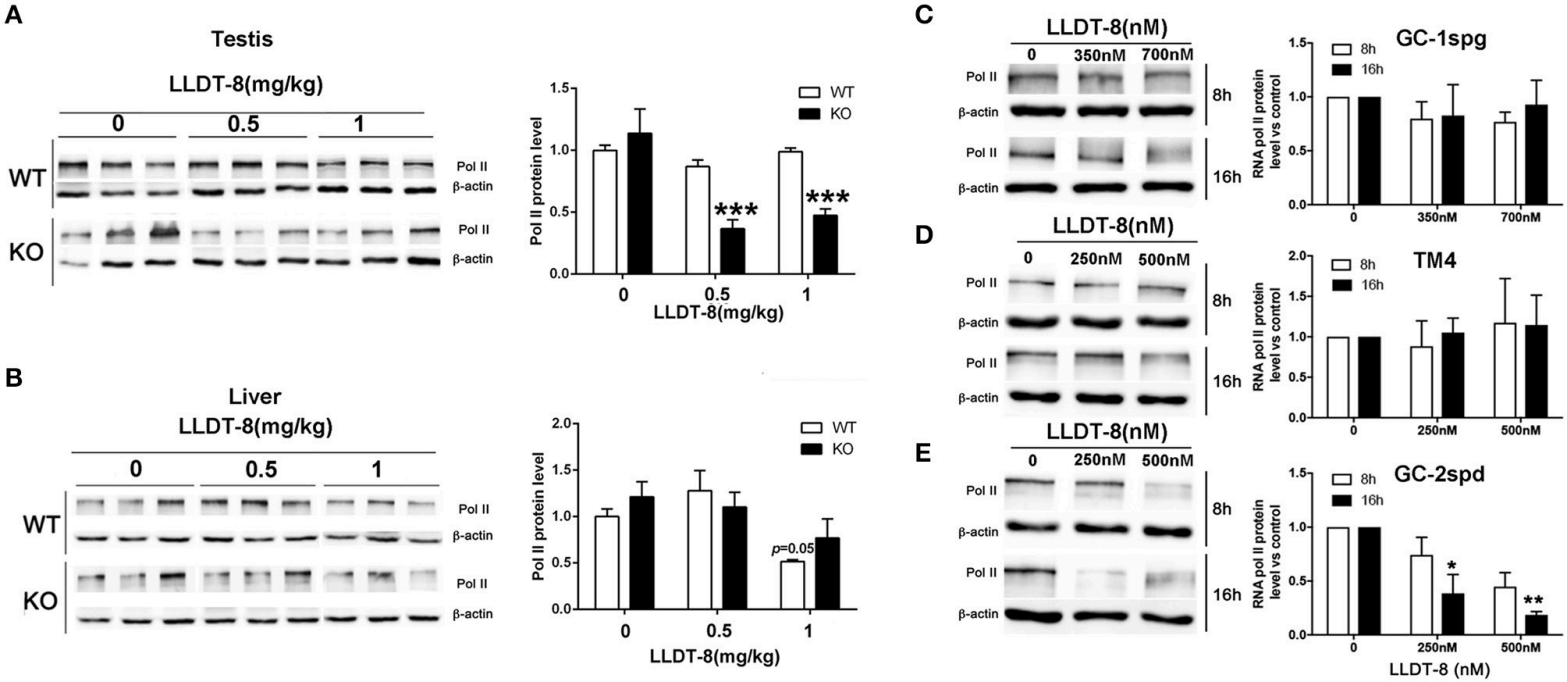
LLDT-8 decreased RNA Pol II *in vivo* and *in vitro*. The protein levels of Pol II were detected by western blot in the testes **(A)**, liver **(B)**, spermatogonia-like GC-1spg cells **(C)**, sertoli-like cells **(D)** and spermatocyte-like cells **(E)**. The protein level was quantified with ImageQuant software. Beta-Actin was used as a loading control. Significant difference was determined by one way ANOVA, mean ± *SD, n* = 3, ^*^p < 0.05, ^**^p < 0.01, ^***^p < 0.001 vs. control group.

In spermatogenesis, the formation of RNA granule and specific transcription activation at some genomic foci (including histone, piRNA.etc) are entirely dependent on RNA Polymerase II (Pol II) (Voronina et al., 2011; Pandey and Pillai, 2014). In KO mice, LLDT-8 decreased Pol II protein in the testes and did not change its level in the livers (Figures 5A,B). In WT mice, the expression of Pol II in the testes and liver did not change significantly (Figures 5A,B). Three immortalized germ cell lines derived from mouse testes, spermatogonia-like GC-1spg, Sertoli-like TM4, and spermatocyte-like GC-2spd were used to evaluate the effects of LLDT-8. LLDT-8 dose- and time-dependently reduced Pol II protein in GC-2spd cells, but not in GC-1spd and TM4 cell lines (Figures 5C–E).

### LLDT-8 did not lessen the expression of ar-related genes in sertoli cells

**Figure 6:**
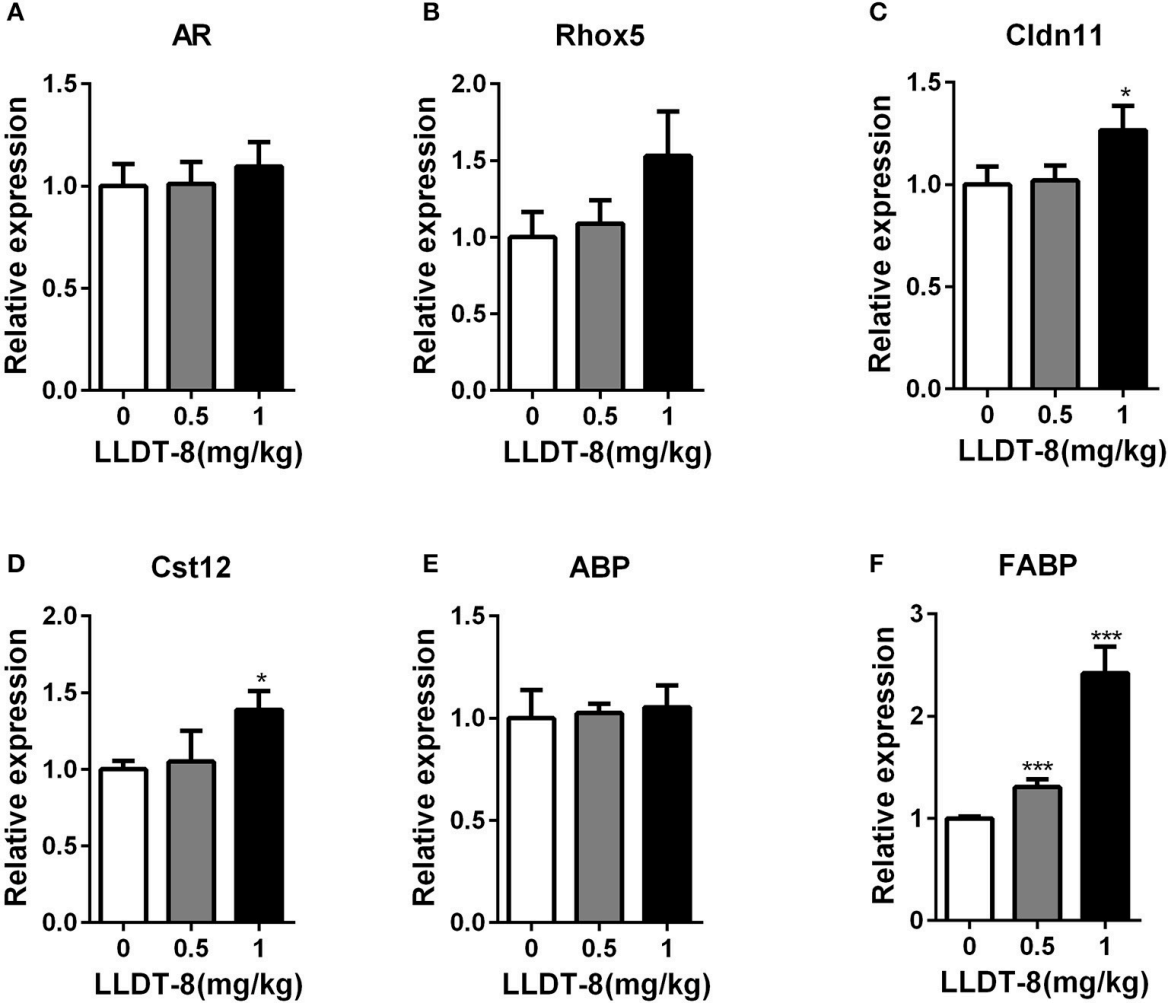
LLDT-8 did not reduce the expression of AR-related genes in sertoli cells. AR **(A)**, Rhox5 **(B)**, Cldn11**(C)**, Cst12 **(D)**, ABP **(E)**, and FABP **(F)** were determined by qPCR. Mean ± *SD, n* = 3, ^*^p < 0.05, ^***^p < 0.001 vs. control group (0 mg/kg, Saline group).

Spermatogenesis is tightly regulated by androgen from Leydig cells and androgen receptor (AR) signaling in Sertoli cells (SCs) (Tan et al., 2005). Here, we selected AR-dependent two genes: Rhox5 and Cldn11 as the key indexs of AR-related signaling in sertoli cells (Willems et al., 2010). We also selected AR-independent three genes: Cst12, ABP and FABP as the indexs of sertoli cell function (Johnston et al., 2004; Li et al., 2005; Tan et al., 2005). LLDT-8 treatment did not reduce the expression of all these genes including AR in the testes of WT mice (Figures 6A–F), while increased the expression of Cldn11, Cst12 and FABP, particularly in 1.0 mg/kg group.

## Discussion

Many efforts have been made to modify triptolide and overcome three major problems: low solubility, toxicity and tissue accumulation (Zhou et al., 2012). One of the outcomes is LLDT-8, (5R)-5-hydroxytriptolide, which shows a favorable safety profile and is well-tolerated in Phase I and II clinical trials in a female population (Supplementary Figure S1). The testicular injury is the major adverse effects in male rodents and dogs (data not shown). As the sole triptolide derivative entering Phase II clinical trial, it's well deserved to clarify the potential contribution of the pharmacokinetics of LLDT-8 to its toxicity, which will be helpful to its Phase III clinical trial in various populations.

Compared to triptolide, LLDT-8 has better pharmacokinetics properties than those of triptolide: lengthened Tmax and non-cumulative property (Xue et al., 2011). Unlike triptolide (Xue et al., 2011), LLDT-8 did not induce hepatotoxicity in the liver of KO mice, while attenuating the liver injury caused by hepatic Cpr knockout (Supplementary Figure S3). Hepatic Cpr knockout inhibits Cyp51 activities, a key enzyme of cholesterol synthesis, interferes hepatic lipid metabolism and induces hepatocytes injuries (Lorbek et al., 2015). Thus, LLDT-8 has a wider therapeutic window.

Hydroxylation via CYP450s and the subsequent glucuronidation, sulfation, and glutathione conjugation is the primary phase I/II elimination pathways of triptolide (Du et al., 2011). We only found positive signal of potential mono-hydroxylated metabolites of LLDT-8 (m/z: 391.1) in WT mice, actively supporting the role of hepatic cyp450s in the hydroxylation of LLDT-8. Under the context of inactivation of hepatic cyp450s, two pathways may be responsible for the clearance of LLDT-8 in KO mice. Firstly, glucuronidation et al phase II metabolism may be responsible for the elimination of LLDT08; Secondly, the remnant 5% the hepatic cyp450s activity via cytochrome b5 as electron donor may also promote the elimination of LLDT-8 in KO mice (Riddick et al., 2013). Hydroxylation decreased the bioactivity of triptolide (Li et al., 2009). It is reasonable to deduce that hydroxylation could subdue the activities of LLDT-8. Our recent work has excluded the potentiality of hydroxylation at position 6 and 20 on LLDT-8. More work is needed to confirm the exact hydroxylation sites on LLDT-8. Additionally, chemical inhibition assay showed that Cyp3a, Cyp2c, Cyp2d and Cyp2e1 may be the main CYP450 isoform responsible for the metabolism of LLDT-8 in the liver of rodents. Here, clomethiazole, an inhibitor of Cyp2e1 seems to completely inhibit the metabolism of LLDT-8, which may be due to its less inhibition on other Cyp450s including Cyp2c and Cyp3a (Stresser et al., 2016).

Serine 139 phosphorylation H2AX (γ-H2AX), a marker for DNA double-strand-breaks, distributes mainly at the leptotene, zygotene, pachytene, diplotene stages of spermatocytes, and indicates the meiotic process, synapsis and XY body formation (a γ-H2AX-positive foci) (Rogakou et al., 1998). RNA polymerase II is essential for the formation of RNA granules in spermatogenesis (Pandey and Pillai, 2014). In this study, LLDT-8 decreased the protein levels of γ-H2AX and RNA pol II in the testes of KO mice. It was a reasonable assumption that the reduction of γ-H2AX and Pol II in the testes of KO mice might be due to the loss of germ cells. Here, we observed a noticeable reduction of γ-H2AX by immunofluorescence, but no apparent loss of spermatocytes in many seminiferous tubules. Thus, we suggested that LLDT-8 may disrupt meiosis by decreasing Pol II and γ-H2AX in KO mice, then induce the spermatocytes apoptosis. In WT mice, immunoblotting did not find a statistically significant reduction of γ-H2AX and Pol II in the testes of WT mice, but the reduction of γ-H2AX in the testes of WT mice was up to 40%. As a derivative of triptolide, LLDT-8 may induced transcription arrest through two mechanisms: (1) LLDT-8 covalently binds XPB, inhibits its DNA-dependent ATPase activity (IC50: 2,900 nM), and leads to the inhibition of Pol II-mediated transcription (Titov et al., 2011); (2) LLDT-8 may trigger CDK7-dependent degradation of Pol II (Manzo et al., 2012). Thus, the reduction of γ-H2AX by LLDT-8 and potential transcription inhibition via XPB binding may mediate the mild but significant damage in the testes of WT mice. Additionally, unlike the normal spermatogenesis *in vivo*, GC-2spd and GC-1spg cell lines are absent of the meiosis process, and the genome is intact under normal culture condition. The increase of γ-H2AX induced by LLDT-8 *in vitro* may be due to the DNA damage caused by LLDT-8 (Supplementary Figure S7).

In our study, histology investigation did not find any abnormality in Leydig cells. Importantly, LLDT-8 treatment could not reduce the expression of AR-dependent and independent genes in sertoli cells. In special, Rhox5, a lead gene for searching SCs and a major androgen response gene, was not influenced by LLDT-8 treatment, strongly supporting that AR-related signaling may be not involved in the testis injury of LLDT-8 (Supplementary Figure S8). We used mouse testis derived GC-1spg (spermatogonia-like), TM4 (Sertoli cell-like) and GC-2spd (spermatocyte-like) *in vitro*. Some previous reports have shown that GC-2spd cells are more sensitive to apoptosis (McKee et al., 2006; Lizama et al., 2011; Qi et al., 2016). Here, the selective reduction of RNA Pol II by LLDT-8 in GC-2spd cells may partly explain this sensitivity difference. However, the detailed mechanisms for this selectivity are still unclear.

In summary, our results indicate that hepatic P450s inactivation can influence the pharmacokinetics and distribution of LLDT-8 *in vivo* and may increase the risk of LLDT-8-induced testicular toxicity. Inactivation or inhibition of P450s is often caused by genetic polymorphism and drug–drug interactions, which contribute to individual differences in xenobiotic metabolism and drug toxicity. Personalized prescription based on the blood concentration could be employed to maximize the therapeutic efficacy of LLDT-8 and reduce its adverse effects in the clinic use, especially in therapies that other drugs with P450 inhibitory property must be utilized along with LLDT-8.

## Author contributions

Conceived and designed the experiments: ChuL, XQ, and JR. Performed the experiments: CY, YL, ML, MG, CheL, HY, ChuL, LS, LM, and CW. Contributed reagents/materials/analysis tools: CY, ChuL, and XQ. Analyzed the data: CY, ChuL, and XQ. Wrote the paper: CY and XQ.

### Conflict of interest statement

The authors declare that the research was conducted in the absence of any commercial or financial relationships that could be construed as a potential conflict of interest.
