# Supplementary figures and images for: Critical Role of Hepatic Cyp450s in the Testis-Specific Toxicity of (5R)-5-Hydroxytriptolide in C57BL/6 Mice
Source: Front Pharmacol. 2017 Nov 21;8:832. doi: 10.3389/fphar.2017.00832 (PMC5702336; doi:10.3389/fphar.2017.00832)

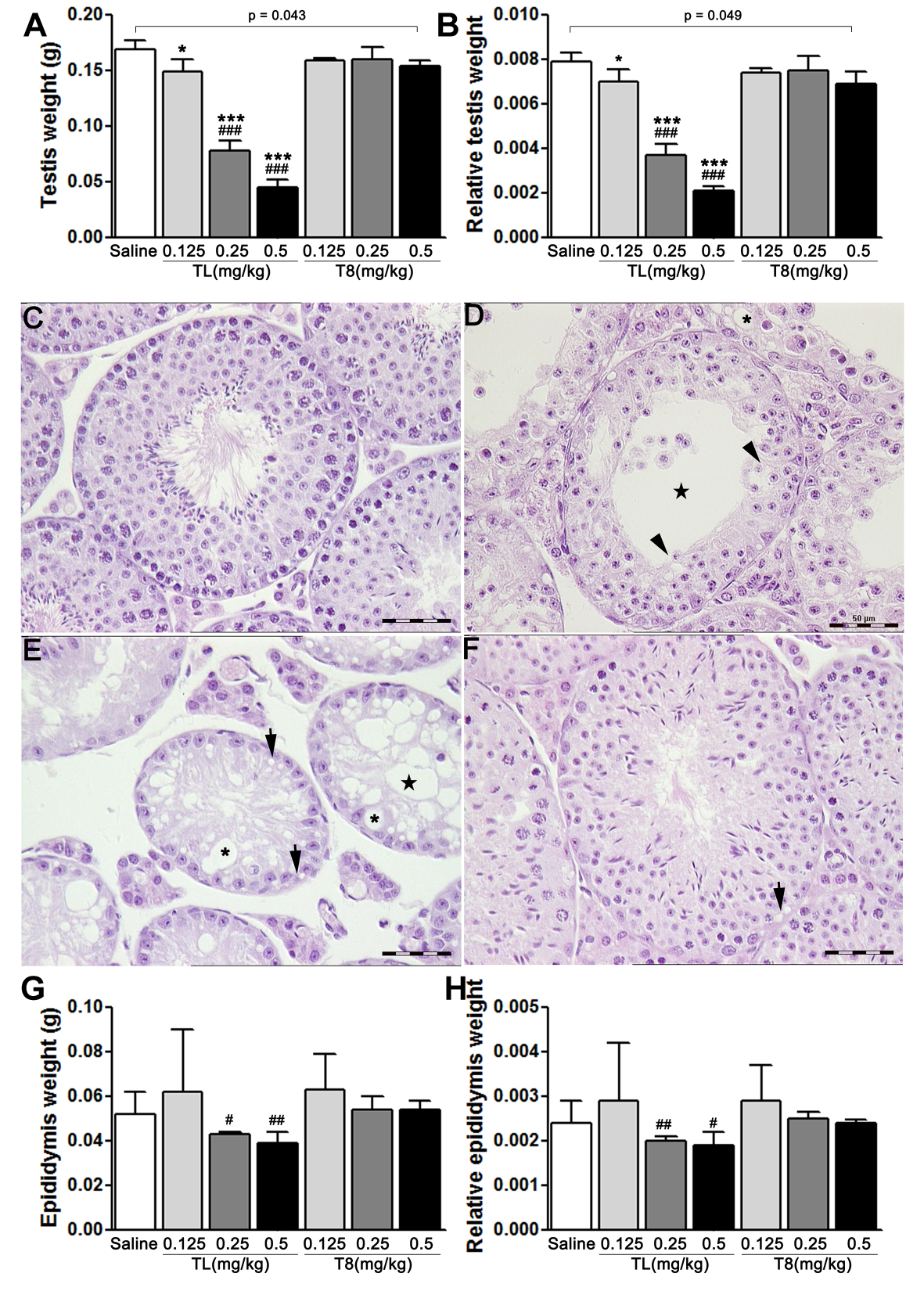

Supplement: Supplementary file 1 [file Image1.TIF]

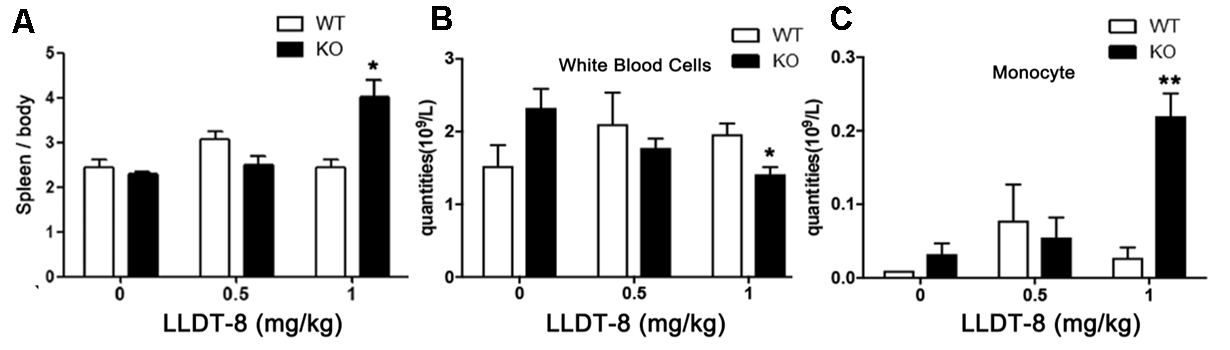

Supplement: Supplementary file 2 [file Image2.TIF]

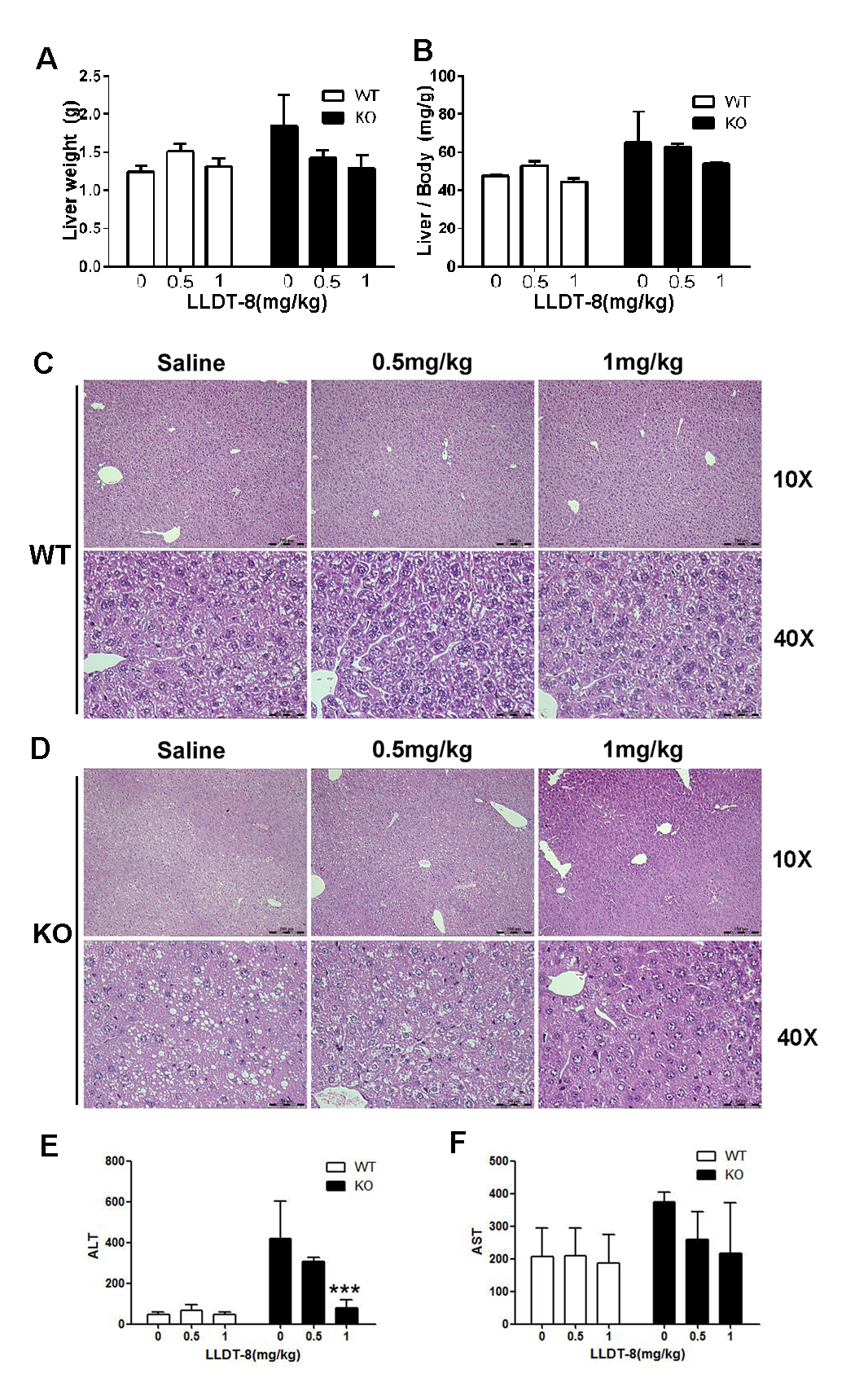

Supplement: Supplementary file 3 [file Image3.TIF]

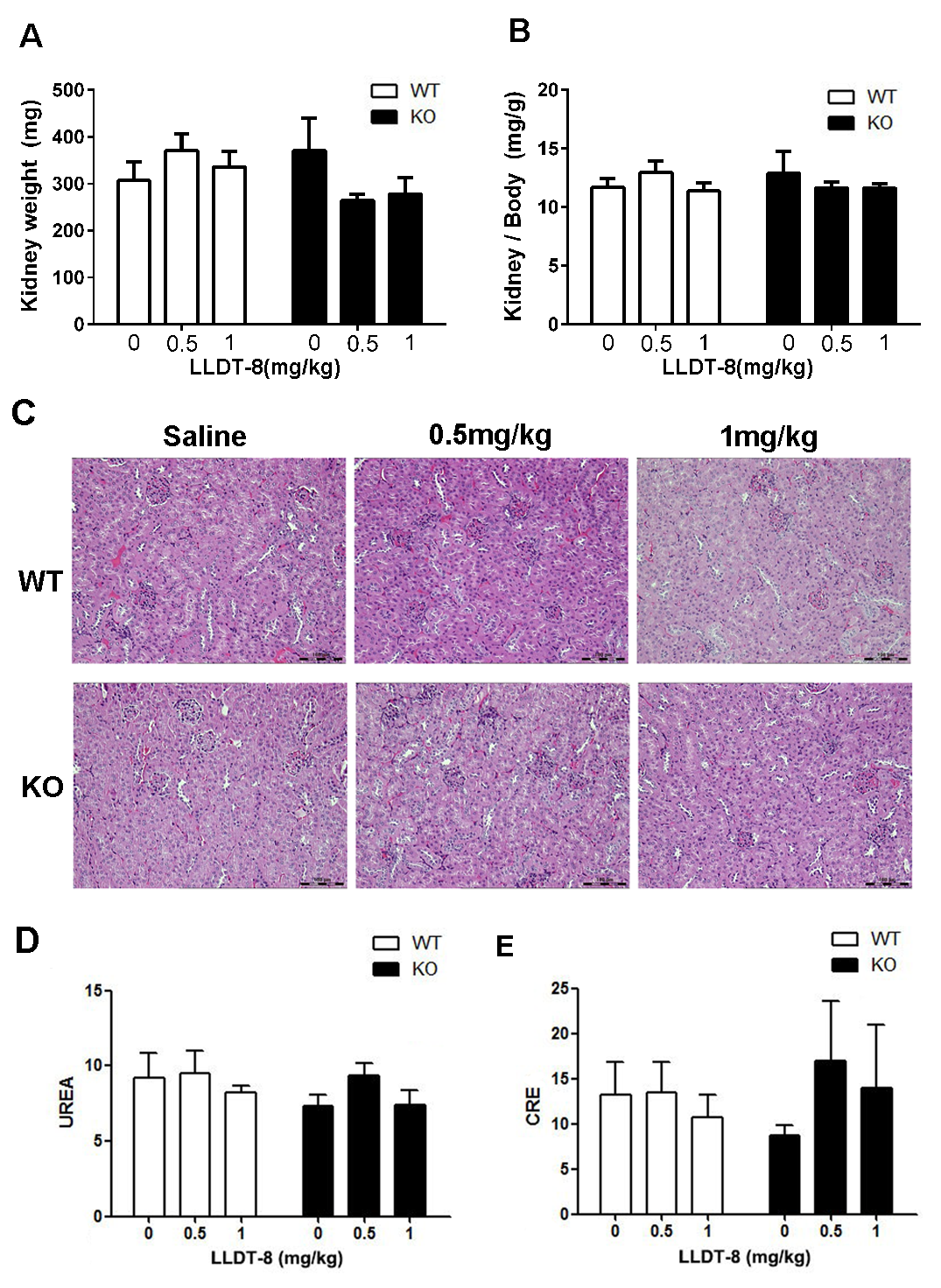

Supplement: Supplementary file 4 [file Image4.TIF]

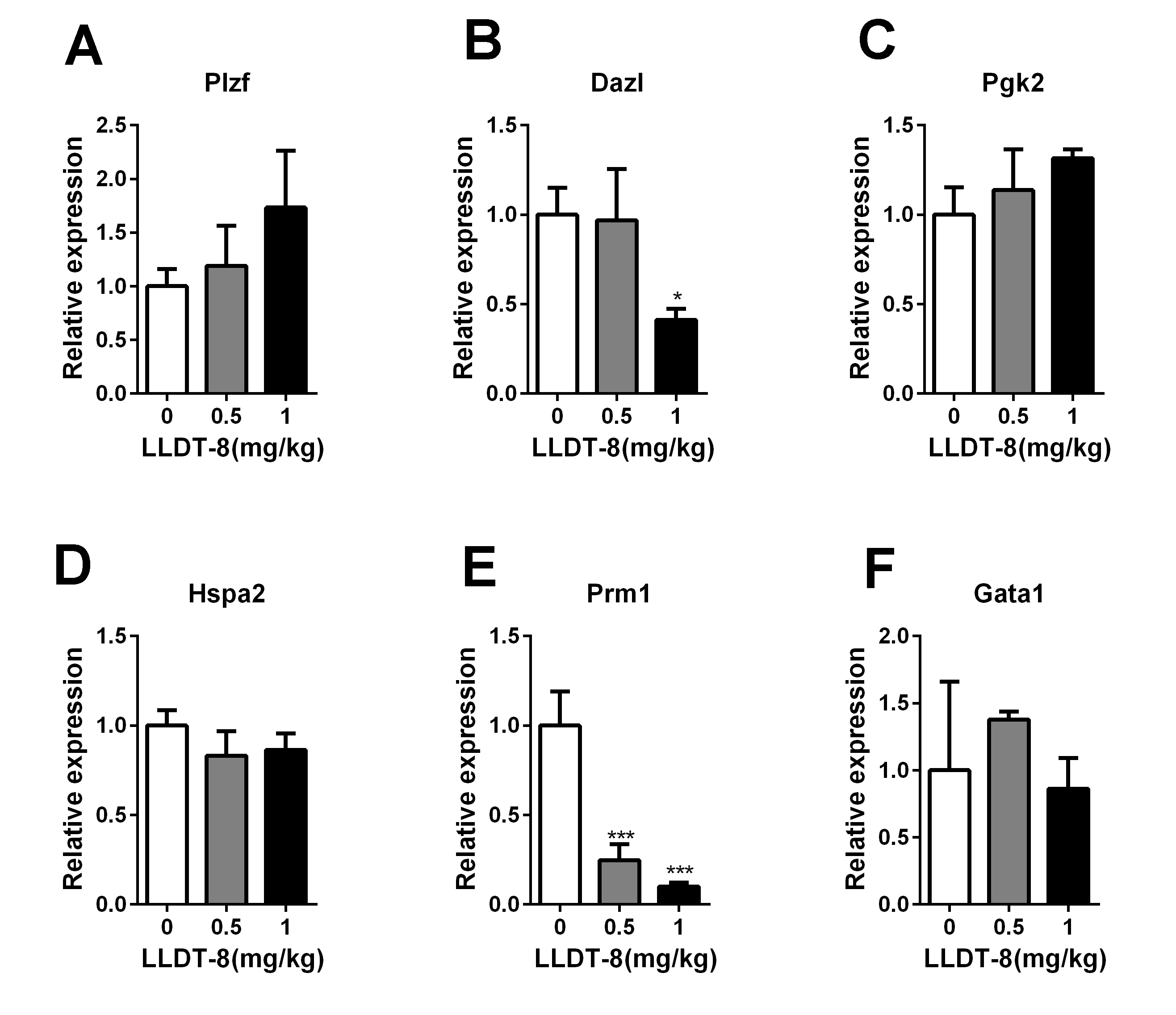

Supplement: Supplementary file 5 [file Image5.TIF]

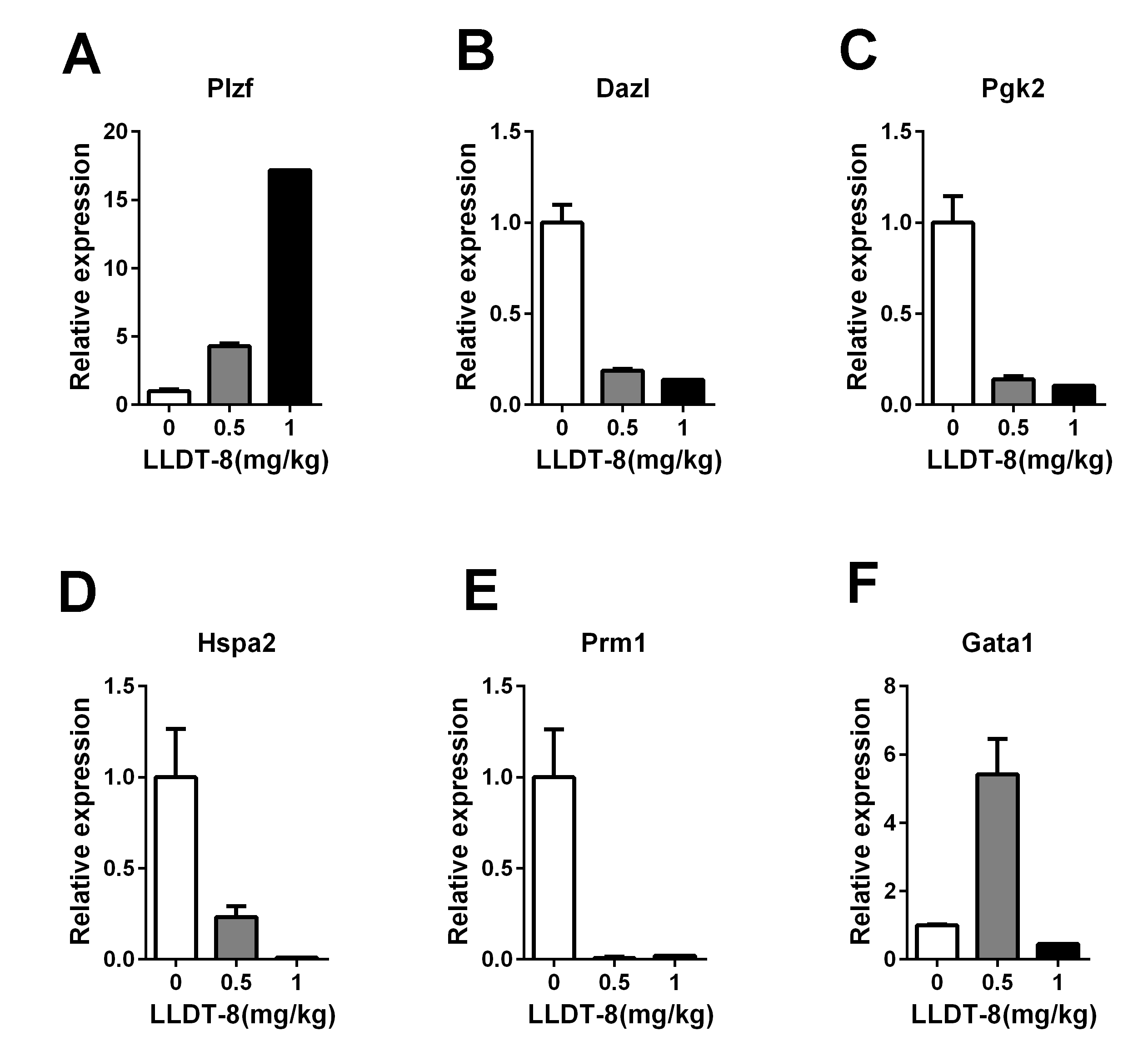

Supplement: Supplementary file 6 [file Image6.TIF]

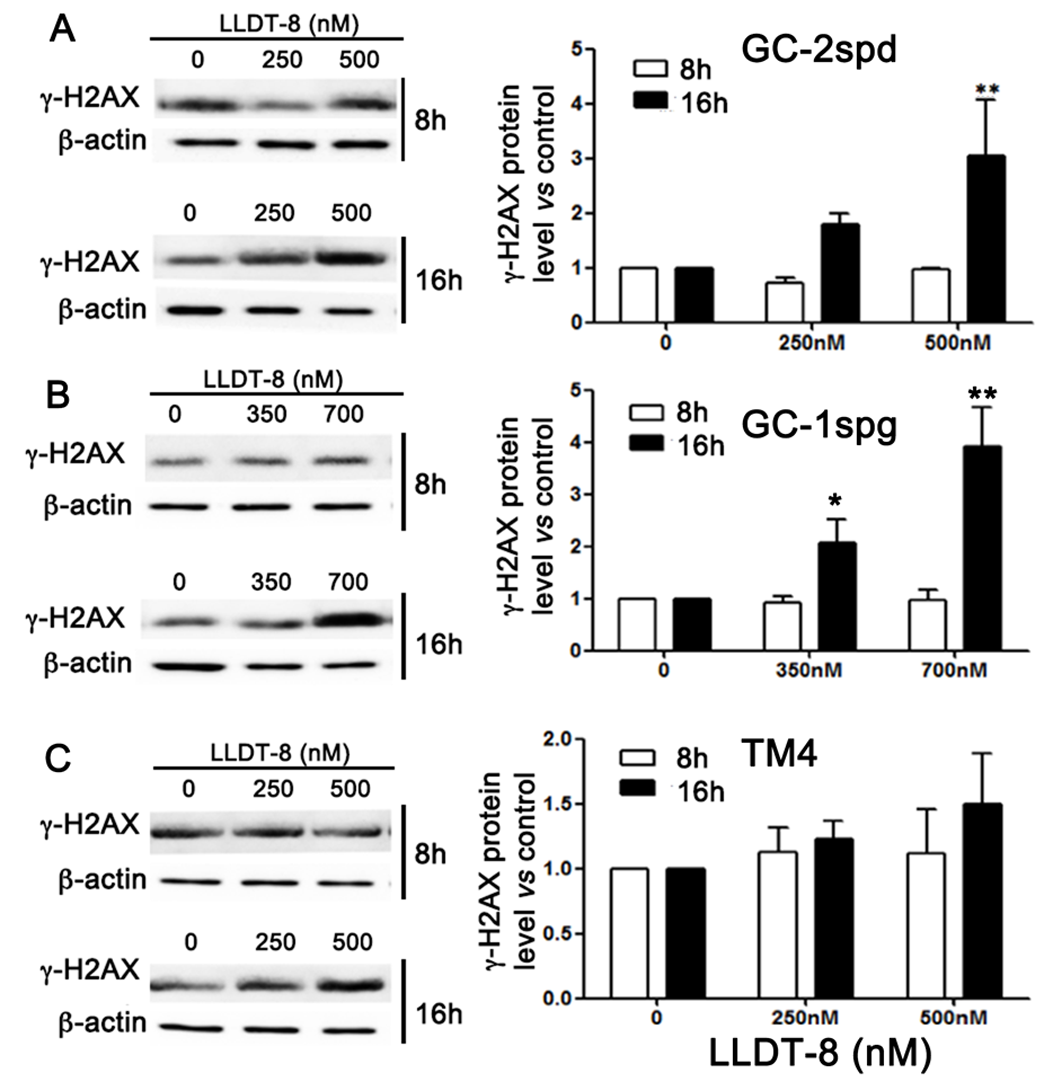

Supplement: Supplementary file 7 [file Image7.TIF]

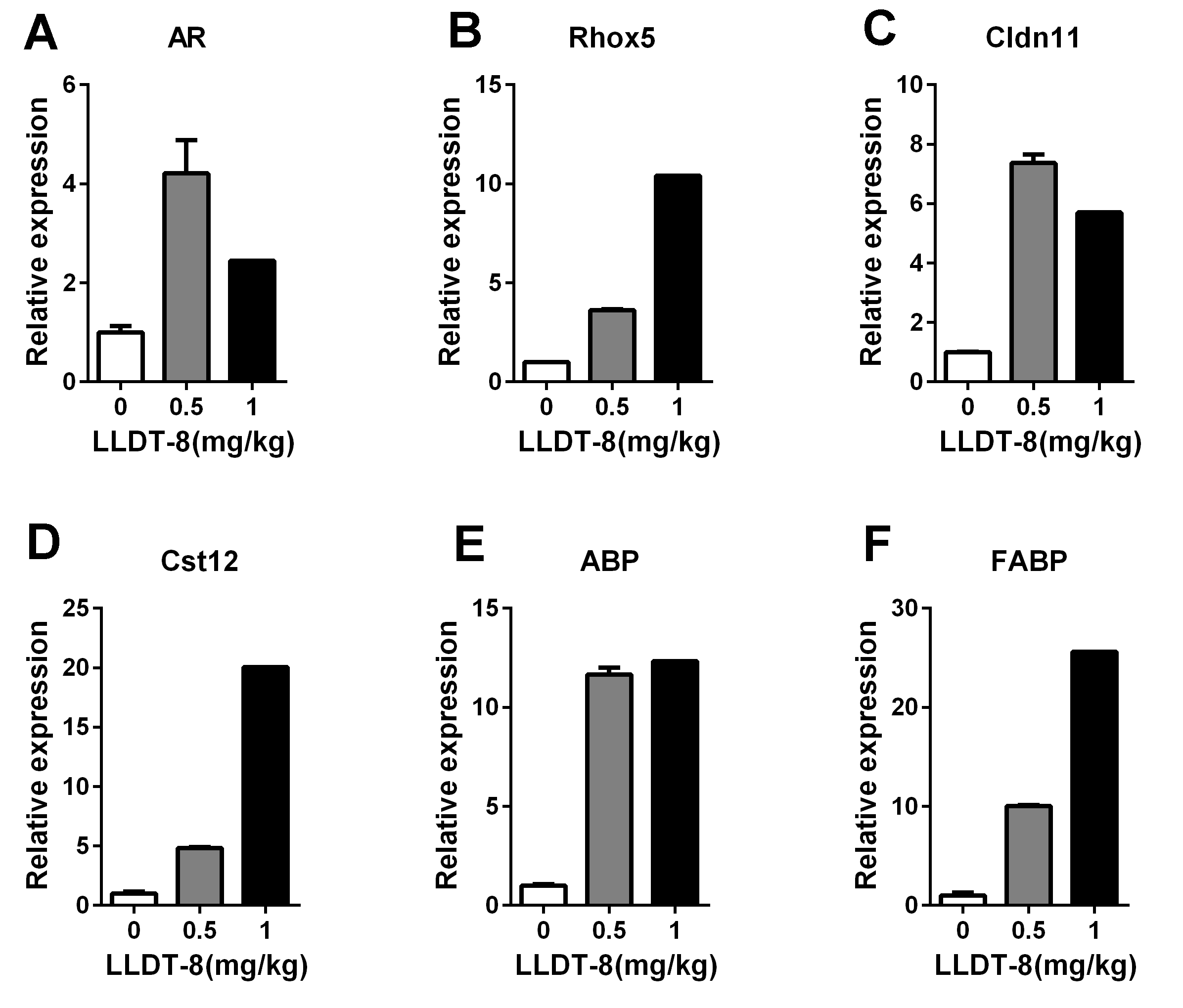

Supplement: Supplementary file 8 [file Image8.TIF]
