# Supplementary material for: Critical Role of Hepatic Cyp450s in the Testis-Specific Toxicity of (5R)-5-Hydroxytriptolide in C57BL/6 Mice
Source: Front Pharmacol. 2017 Nov 21;8:832. doi: 10.3389/fphar.2017.00832 (PMC5702336; doi:10.3389/fphar.2017.00832)
Supplement: Supplementary file 9 [file SupplementaryFigureLegends.docx]

**Supplementary materials and methods**

**Testicular toxicity comparison between triptolide and LLDT-8**

For toxicity experiments, mice (n = 3 for each group) were treated with tritpilide (0.125, 0.25, 0.5mg/kg), LLDT-8 (0.125, 0.25, 0.5mg/kg) consecutively administered by gavage for 15 days. The control group received saline. Mice were sacrificed on the 15^th^ day post-administration and blood, liver, kidney, spleen, testis and epididymis were collected. The main lobe of liver, kidney, spleen and epididymis were fixed in 10% neutral buffered formalin for histological examination, the left testicle was fixed in Davidson’s buffer for 16 hours followed by 10% neutral buffered formalin. Tissue sections were stained with hematoxylin and eosin (H&E). Remaining tissues were stored at -80^°^C for RNA and protein extraction.

Sera were assayed for Urea, creatine (CRE), alanine aminotransferase (ALT), and aspartate transaminase (AST) using an automatic HITACHI Clinical Analyzer Model 7080 (Hitachi High-Technologies Corporation, Tokyo, Japan). The intercoefficients of variability in this assay were 1.1% (ALT), 0.9% (AST), 1.2% (Urea) and 1.4% (CRE). The intracoefficients of variability in this assay were 2.0% (ALT), 0.9% (AST), 1.2% (Urea) and 5.1% (CRE).

**Note: To validate our conclusion, we detected mRNA levels of germ cell markers(Plzf, Dazl, Pgk2, Hspa2, Prm1 and Gata1) and AR-related genes in sertoli cells(AR，Pem, FABP, PDGF-A, transferrin, ABP, Cystatin-TE, Claudin 11, FSHR and AMH). It should be noted that the animal number for qPCR in KO groups was not enough (The animal number of Saline group was Two, the number of 0.5mg/kg group was Two, the number of 1mg/kg group was One, Supplementary Figure S5 and S7), because of we preferably satisfied the need of histology and protein extraction (Figure 1, 2, 4 and 5), which were relatively helpful to elaborate our conclusion.**

**Figure legends**

**Supplementary Figure S1 Comparison of testicular toxicity between triptolide(TL) and LLDT-8.** Mice (n=3) were weighted before sacrificed on the 15^th^ day post-administration of triptolide, LLDT-8 or saline. The testes were collected and weighted. **(A)** Testes weights of mice in each group; **(B)** Testes relative weight (testis absolute weight *vs* body weight) was shown here; **(C-F)** The left testicle of each mouse was stained with H&E (×10, ×40); The testis in saline group had normal spermatids development **(C)**, while after triptolide treatment for 15days, both 0.25mg/kg triptolide **(D)** and 0.5mg/kg triptolide **(E)** induced severe testicular injury including reduction of germinal layers **(star)**, vacuolar degeneration **(asterisk)** and abnormally developed spermatocytes and spermatids **(arrow)**; however, LLDT-8 treatment with 0.5mg/kg did not obviously alter the normal morphology of testes **(F)**. **(G-H)** Comparison of epididymis weight between triptolide and LLDT-8. Significant difference was determined by one-way Anova, mean ± SD. * *p*<0.05, *** *p*<0.001 *vs* Saline group. # *p*<0.05, ## *p*<0.01, ### *p*<0.001 *vs* T8 group at the same dosage.

**Supplementary Figure S2 LLDT-8 induced hematological change and enlarged spleen in KO mice. (A)** spleen relative weight (spleen absolute weight *vs* body weight); **(B)** The total number of white blood cells. **(C)** The total number of monocytes. Significant difference was determined by one way Anova, mean ±SD, n = 3, * *p*<0.05, ** *p*<0.01 *vs* Saline group.

**Supplementary Figure S3 LLDT-8 did not aggravate liver injury. (A)** liver weights of WT and KO mice; **(B)** livers relative weight (liver absolute weight *vs* body weight) was shown here; The liver of WT **(C)** and KO **(D)** mouse was stained with H&E (×10, ×40); **(E, F)** Plasma ALT and AST of each mouse. Significant difference was determined by one-way Anova, mean ±SD, n=3, *** *p*<0.001 *vs* Saline group.

**Supplementary Figure S4 LLDT-8 did not aggravate kidney injury.** **(A)** kidney weights of WT and KO mice; **(B)** kidneys relative weight (liver absolute weight *vs* body weight) was shown here; **(C)** The kidneys of each mouse were stained with H&E (×20); Plasma Urea **(D)** and creatine **(E)** of each mouse were shown here. Significant difference was determined by one-way Anova, mean ±SD, n=3.

**Supplementary Figure S5 LLDT-8 affected germ cell specific differentiation markers gene expression in WT mice.** The transcript levels of markers for germ cellspecific differentiation were detected by qPCR. **(A)**Plzf: a marker for germ stem cells and type A spermatogonia; **(B)** Dazl: a marker for type B spermatogonia and primary spermatocytes; **(C)** Pgk2: a marker for meiotic spermatocytes; **(D)** Hspa2: a marker for post-meiotic spermatocytes and spermatids; **(E)** Prm1: a marker for spermatids; **(F)** Gata1: a marker for sertoli cells; Significant difference was determined by one way Anova, mean ±SD. * *p*<0.05, *** *p*<0.001.

**Supplementary Figure S6 LLDT-8 affected germ cell specific differentiation markers gene expression in KO mice.** The transcript levels of markers for germ cellspecific differentiation were detected by qPCR. **(A)** Plzf; **(B)** Dazl; **(C)** Pgk2; **(D)** Hspa2; **(E)** Prm1; **(F)** Gata1; Bars represent the mean ± SD. The animal number for the qPCR was not enough (the number of Saline group was Two, the number of 0.5mg/kg group was Two, the number of 1mg/kg group was One).

**Supplementary Figure S7 LLDT-8 did not decrease the protein levels of γ-H2AX in three Germ-cell lines.** The effects of LLDT-8 on the expression of γ-H2AX was investigated in spermatocyte-like GC-2spd cells **(A)**, spermatogonia-like cells **(B)** and sertoli-like cells **(C)** after 8h/16h treatment of LLDT-8 at indicated concentration. The protein level was quantified with ImageQuant software. β-actin was used as a loading control. Significant difference was determined by one-way Anova, Bars represent the mean ± SD, n = 3, * *p*<0.05, ** *p*<0.01 vs control group (0 nM).

**Supplementary Figure S8 LLDT-8 did not reduce the expression of AR-related genes in sertoli cells.** AR (A), Rhox5 (B), Cldn11 (C), Cst12 (D), ABP (E) and FABP(F) were determined by qPCR. Bars represent the mean ± SD. The animal number for the qPCR was not enough (the number of Saline group was Two, the number of 0.5mg/kg group was Two, the number of 1mg/kg group was One).
